# Phosphorylation of Daxx by ATM Contributes to DNA Damage-Induced p53 Activation

**DOI:** 10.1371/journal.pone.0055813

**Published:** 2013-02-06

**Authors:** Jun Tang, Trisha Agrawal, Qian Cheng, Like Qu, Michael D. Brewer, Jiandong Chen, Xiaolu Yang

**Affiliations:** 1 Department of Cancer Biology and Abramson Family Cancer Research Institute, Perelman School of Medicine, University of Pennsylvania, Philadelphia, Pennsylvania, United States of America; 2 Department of Molecular Oncology, Moffitt Cancer Center, Tampa, Florida, United States of America; Wayne State University School of Medicine, United States of America

## Abstract

p53 plays a central role in tumor suppression. It does so by inducing anti-proliferative processes as a response to various tumor-promoting stresses. p53 is regulated by the ubiquitin ligase Mdm2. The optimal function of Mdm2 requires Daxx, which stabilizes Mdm2 through the deubiquitinase Hausp/USP7 and also directly promotes Mdm2’s ubiquitin ligase activity towards p53. The Daxx-Mdm2 interaction is disrupted upon DNA damage. However, both the mechanisms and the consequence of the Daxx-Mdm2 dissociation are not understood. Here we show that upon DNA damage Daxx is phosphorylated in a manner that is dependent on ATM, a member of the PI 3-kinase family that orchestrates the DNA damage response. The main phosphorylation site of Daxx is identified to be Ser564, which is a direct target of ATM. Phosphorylation of endogenous Daxx at Ser564 occurs rapidly during the DNA damage response and precedes p53 activation. Blockage of this phosphorylation event prevents the separation of Daxx from Mdm2, stabilizes Mdm2, and inhibits DNA damage-induced p53 activation. These results suggest that phosphorylation of Daxx by ATM upon DNA damage disrupts the Daxx-Mdm2 interaction and facilitates p53 activation.

## Introduction

Cells carrying an activated oncogene, damaged genome, or other cancer-promoting alterations are normally prevented from replicating through an elaborate tumor suppression network. A central hub of this network is p53 [1], [2]. p53 is a transcription factor that controls the expression of a large number of genes involved in cell cycle arrest, apoptosis, and senescence [3], [4]. p53 also has transcription-independent functions in the induction of cytochrome *c* release from mitochondria [5], [6] and the inhibition of glucose metabolism and biosynthesis [7], [8].

The potent anti-proliferative function of p53 makes its regulation a principal issue within animal cells. In unstressed cells, p53 is a short-lived protein due to its rapid ubiquitination and degradation in the 26S proteasome. p53 degradation is largely mediated by Mdm2 (mouse double minute, also known as Hdm2 in humans), a RING domain-containing E3 ubiquitin ligase [9], [10], [11]. The inhibition of Mdm2 under stress conditions enables p53 to stabilize. p53 stabilization induced by DNA damage specifically is dependent on ATM (ataxia telangiectasia mutated) [12], which orchestrates the cellular response to DNA double strand breaks by phosphorylating a wide range of substrates. ATM and its downstream kinase Chk2 phosphorylate p53 in the Mdm2-interacting N-terminal region (at Ser15 and Ser20, respectively), which weakens the interaction of p53 with Mdm2 [13], [14], [15], [16]. However, targeted mutations of one or both of the corresponding sites in murine p53 led to only modest defects in p53 activation [17], [18], [19], indicating that other mechanisms downstream of ATM may also contribute to inactivation of Mdm2.

A critical regulator of Mdm2 is Daxx (death domain-associated protein) [20]. In unstressed cells, Daxx binds simultaneously to Mdm2 and the deubiquitinase Hausp (herpesvirus-associated ubiquitin-specific protease; also known as USP7), mediating the stabilizing effect of Hausp on Mdm2 [20]. In addition, Daxx directly stimulates Mdm2’s ubiquitin E3 ligase activity towards p53 [20]. In cells challenged with DNA damaging agents, the Mdm2-Daxx interaction is disrupted in an ATM-dependent manner, which is followed by p53 activation [20]. The Mdm2-Daxx interaction is also disrupted by the tumor suppressor RASSF1A [21]. The mechanism by which DNA damage signals dissociate Daxx from Mdm2 and its consequences on Mdm2 and p53 remain unclear. Previously, it was reported that ATM phosphorylates Mdm2 at Ser395 [22]. A recent study identified additional Ser residues at the Mdm2 C-terminus as ATM target sites. The phosphorylation of these Ser residues decreases Mdm2 activity in a redundant manner with each other and with the phosphorylation at Ser395 [23]. However, a phospho-mimic mutant of Mdm2 (S395D) does not dissociate Mdm2 from Daxx [20], making it possible that Daxx may be another target of ATM. The objective of this study was to investigate whether ATM phosphorylates Daxx and, if so, whether this phosphorylation influences the Daxx-Mdm2 interaction and DNA damage-induced p53 activation.

## Materials and Methods

### Antibodies and plasmids

Antibodies for the following proteins/epitopes were purchased from the indicated sources: actin, tubulin, and Flag (mouse monoclonal, M2, free and conjugated to beads, and rabbit polyclonal) (Sigma); ATM (Ab-3) and Mdm2 (Ab-1 and Ab-3) (Calbiochem); Daxx (M-112), p53 (DO-1), and PML (Santa Cruz Biotechnology); phosphorylated ATM/ATR consensus site (pS/T-Q) (#2851, Cell Signaling); GFP (JL-8, Clontech); Hausp/USP7 (A300, Bethyl Laboratories, Inc.); HA conjugated to horseradish peroxidase (Roche). Antibody specific to Phospho-Daxx Ser564 was made by Invitrogen using peptide PEELTLEEESPVpSQLFELEIEA.

Plasmids encoding HA- or Flag-tagged Mdm2 and Daxx for transient transfection were made in pRK5, and plasmids encoding Flag-tagged Daxx for stable infection were made in the retroviral vector pBabe-puro. They were either previously described (14), or generated for this study by PCR and confirmed by sequencing. The Daxx-EGFP plasmid was made in pEGFP-C1 (Clontech). ATM and ATM KD expression plasmids were kindly provided by Dr. M. B. Kastan.

### Cell Culture

All cells were obtained from the ATCC. H1299 cells were grown in RPMI-40 media and all the other cell lines in Dulbecco’s modified Eagle’s medium, supplemented with 10% fetal bovine serum and 1% penicillin/streptomycin. For generating Daxx and control stable cell lines, retroviral constructs for Flag-Daxx and Flag-Daxx S564A, as well as the parental vector pBabe-puro, were separately transfected into either Phoenix cells along with the retroviral packaging vector pCL-Ampho, or HEK293T cells along with pcgp (which encodes gag pol) and pHIT 456 (which encodes retroviral envelope). 48-72 h after transfection, the retrovirus-containing medium was used to infect U2OS or MCF-7 cells in the presence of 8 µg/mL polybrene. The infected cells were selected in the presence of 2 µg/ml puromycin for 4-5 days.

### Immunoprecipitation and Western blot

Transfections were carried out using Lipofectamine 2000 (for DNA) or RNAiMAX (for siRNA) (Invitrogen) according to the manufacturer’s instructions. 24 h after transfection, cells were lysed in IP lysis buffer (50 mM HEPES at pH 8.0, 150 mM NaCl, 0.5% Triton X-100, 0.5% NP-40, 100 mM NaF, 1 mM PMSF, 1 mM DTT, 1X complete protease cocktail, and 10% glycerol). Flag-Daxx or Flag-Mdm2 was immunoprecipitated with anti-Flag mAb beads and analyzed by western blot with anti-Daxx antibody (1:5,000), anti-phosphorylated ATM/ATR consensus site (pS/T-Q) antibody (1:500), anti-Flag antibody (mouse or rabbit, 1:5,000), or anti-HA antibody (1:5,000).

### Kinase Assay

Flag-tagged ATM wild-type (WT), ATM KD, Daxx, and Daxx S564A were separately expressed in 293T cells and purified using M2 beads as previously described [23], [24]. Daxx or Daxx S564A was incubated with ATM or ATM KD at 30°C for 1 hour in kinase buffer (50 mM HEPES, pH 7.5, 150 mM NaCl, 20 mM MnCl_2_, and 10% Triton X-100) containing 2 μM ATP and 10 μCi γ-^32^P-ATP. Samples were fractionated on a 7.5% SDS-PAGE gel and detected by autoradiography and western blot.

### Quantitative RT-PCR analysis

Total RNA was isolated from cells using TRIzol (Invitrogen). Reverse Transcription was performed using the First Strand cDNA Synthesis Kit (Marligen Biosciences). A Taqman Gene Expression Assay (Applied Biosystems) with validated human p21 (Hs00355782_m1) and 18s rRNA (4333760F) primers/probe sets (Applied Biosystems) were used for qPCR and analyzed.

## Results

### Daxx is phosphorylated at Ser564 in response to DNA damage

**Figure 1:**
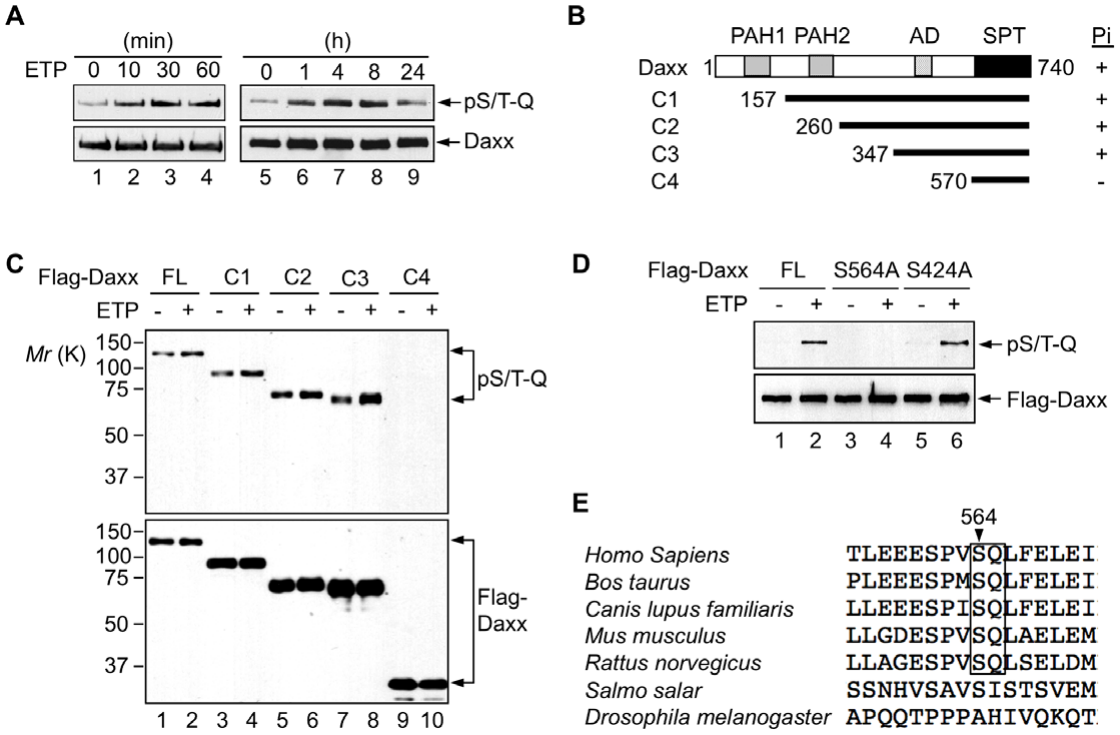
Daxx is phosphorylated at Ser564 in response to DNA damage. (A) Flag-Daxx is phosphorylated upon DNA damage. p53-deficient H1299 cells were transiently transfected with Flag-tagged Daxx. 24 h later, the cells were treated with 10 μM etoposide (ETP) for the indicated durations. Cells were lysed and Flag-Daxx was immunoprecipitated with anti-Flag mAb (M2) beads and analyzed by western blot with antibodies against Daxx or phosphorylated ATM substrate consensus site (pS/T-Q). (B) Schematic representation of full length Daxx and its N-terminal deletion mutants. PAH, paired amphipathic alpha helices domain. AD, acidic-rich domain. SPT, Ser/Pro/Thr-rich domain. The amino acids in full length Daxx and in the N-terminus of each deletion mutant, and phosphorylation (Pi) of these mutants are indicated. (C) Phosphorylation of Daxx deletion mutants in response to DNA damage. H1299 cells expressing full-length (FL) Daxx and each of the deletion mutants were treated with ETP for 1 h. Phosphorylation of these proteins was analyzed as in (A). Exogenous Daxx phosphorylation existing before DNA damage was observed in some experiments, but not others. (D) Phosphorylation of Daxx at Ser564. Phosphorylation of Daxx, Daxx S424A, and Daxx S564A upon DNA damage was analyzed as in (c). (E) Alignment of the human Daxx (gi|48146287) sequence around Ser564 with the corresponding Daxx sequences from *Bos taurus* (gi|296474559), *Canis lupis familiaris* (gi|55956960), *Mus musculus* (gi|2253707), *Rattus norvegicus* (gi|18148939), *Salmo salar* (gi|148362139), and *Drosophila melanogaster* (gi|54144924). Alignment was run using Clustal 2.1 [27].

To examine the possibility that upon DNA damage Daxx is phosphorylated at an ATM consensus site(s), we expressed Flag-tagged Daxx in the human lung cancer cell line H1299 and treated these cells with the genotoxic drug etoposide. An antibody that recognizes the phosphorylated, ATM substrate consensus sequence X-Ser/Thr-Gln (where X is a hydrophobic residue) was then used to detect Daxx phosphorylation. Daxx phosphorylation was observed as early as ten minutes after etoposide treatment, and remained for over eight hours (Figure 1A). To identify the phosphorylation site(s) on Daxx, we deleted Daxx amino acid residues progressively from the N-terminus (Figure 1B). Deletion up to the amino acid residue 347 had no apparent effect on Daxx phosphorylation, but further deletion to the amino acid residue 570 completely abolished it (Figure 1C), suggesting that the major ATM phosphorylation site(s) is between residues 347 and 570. A survey of this region revealed two ATM substrate consensus sequences: MAS^424^QG and PVS^564^QL. We mutated Ser424 and Ser564 individually to Ala. The Ser564-to-Ala (S564A) mutation, but not the Ser424-to-Ala (S424A) mutation, eliminated Daxx phosphorylation (Figure 1D), suggesting that Ser564 is the major ATM-dependent phosphorylation site. The VS^564^Q and the flanking sequences are highly conserved in Daxx proteins from various mammalian species, but not in fish or flies (Figure 1E).

### Phosphorylation of endogenous Daxx upon DNA damage

**Figure 2:**
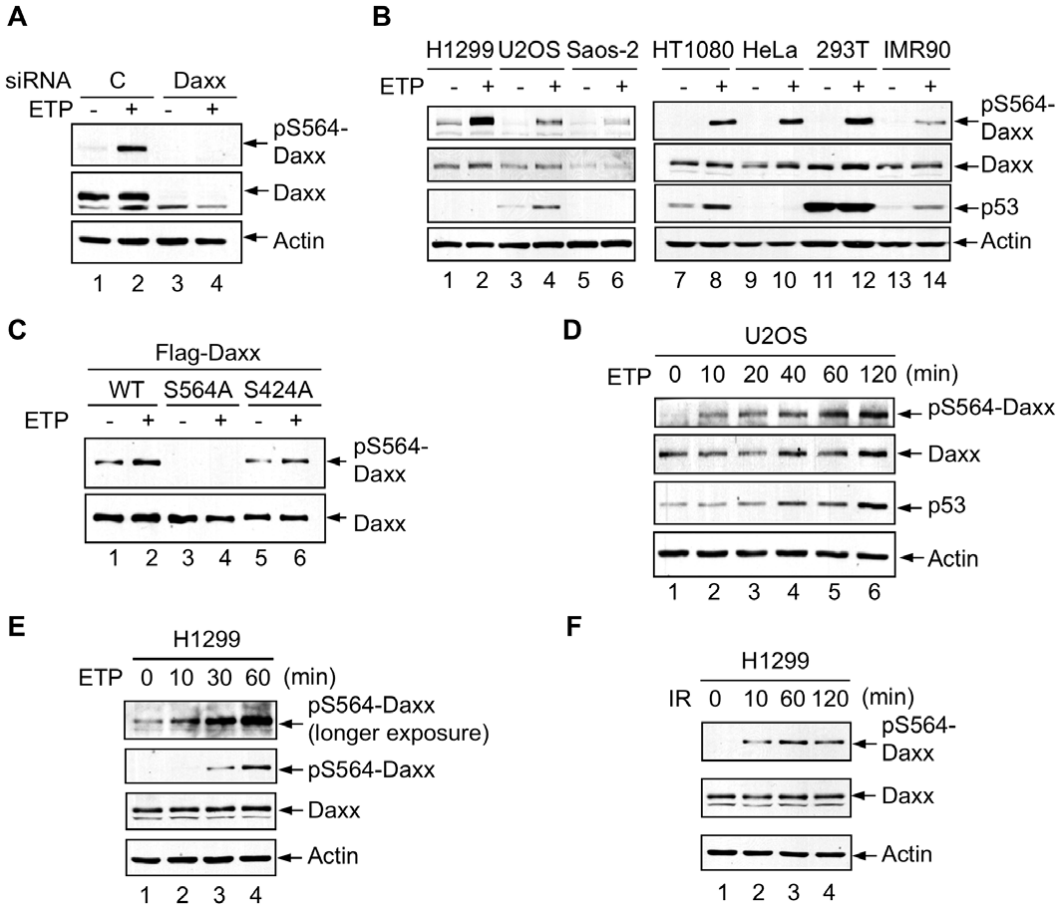
Phosphorylation of endogenous Daxx upon DNA damage. (A) U2OS cells were transfected with control or Daxx siRNA and treated with ETP for 1 h. Cell lysates were analyzed by western blot using phospho-specific Daxx antibody, pS564-Daxx. (B) Phosphorylation of endogenous Daxx in multiple cell lines treated with and without etoposide for 1 h. Cell lysates were analyzed using antibodies against pS564-Daxx, Daxx, p53, and actin. (C) Western blot analysis of H1299 cells transfected with wild-type (WT) Daxx, Daxx S564A, or Daxx S424A and treated with ETP for 1 h. (D and E) U2OS (D) and H1299 (E) cells treated with ETP for the indicated time periods were analyzed by western blot. (F) H1299 cells were exposed to 10 Gy of ionizing radiation (IR) and cultured for the indicated time periods before analysis of Daxx phosphorylation.

To confirm the phosphorylation of endogenous Daxx upon DNA damage, we raised an antibody that recognized the phosphorylated Ser564 of Daxx (pS564-Daxx). This antibody detected phosphorylation of endogenous Daxx in the p53-wild-type U2OS cells upon DNA damage (Figure 2, A and B). It also detected phosphorylation of exogenous wild-type Daxx and Daxx S424A, especially when cells were treated with etoposide (Figure 2C). However, the phosphorylation signal disappeared when endogenous Daxx was knocked down in U2OS cells by small interfering RNA (siRNA) (Figure 2A), or when Daxx S564A was expressed (Figure 2C). These observations underscored the specificity of the anti-pS564-Daxx antibody. Using this antibody, we observed DNA damage-induced phosphorylation of endogenous Daxx in a range of other tumor or primary cell lines including the p53-wild-type HT1080 and IMR90 cells and the p53-deficient H1299, Saos-2, HeLa, and 293T cells (Figure 2B), indicating its generality and its independence of p53 status. A time course analysis showed that phosphorylation of endogenous Daxx became detectable within ten minutes after treatment with either etoposide (Figure 2, D and E) or ionizing radiation (Figure 2F). In the p53 wild-type cells, Daxx phosphorylation preceded an increase in p53 levels (Figure 2D).

### Daxx is phosphorylated by ATM *in vivo* and *in vitro*

**Figure 3:**
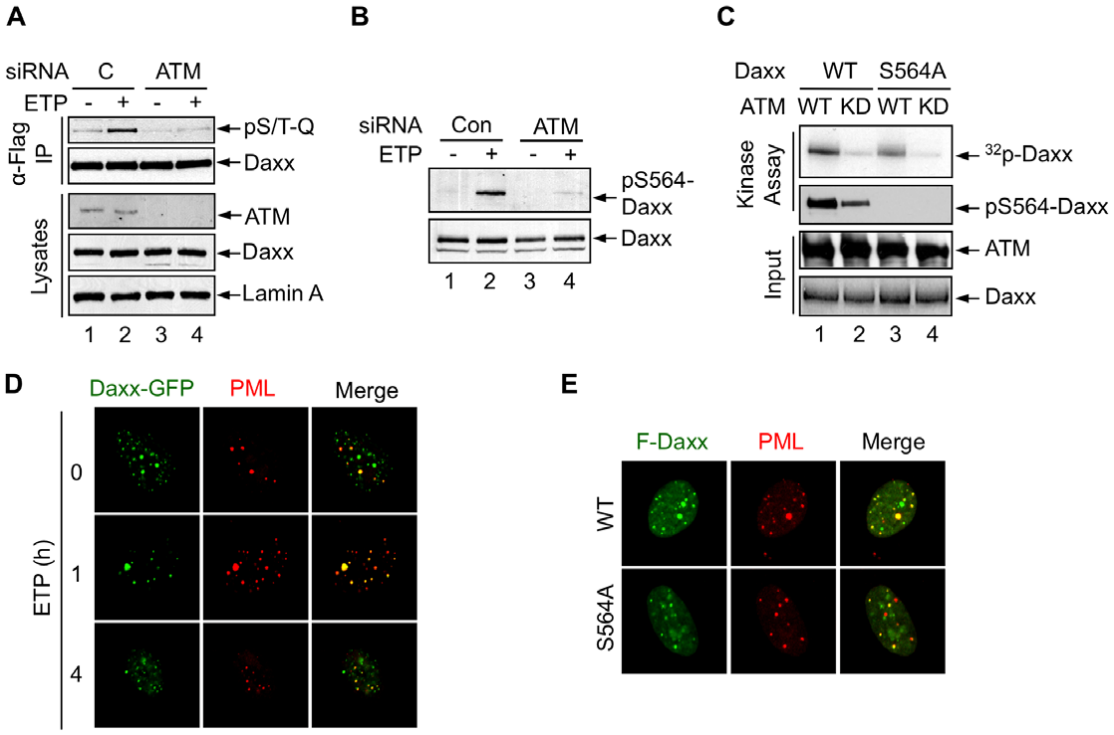
Daxx is phosphorylated by ATM *in vivo* and *in vitro*. (A) H1299 cells treated with a control (C) siRNA or ATM siRNA were transfected with Flag-Daxx and treated with etoposide. Cell lysates and immunoprecipitated Flag-Daxx were examined by western blot using anti-ATM, lamin A, anti-Daxx, and anti-pS/T-Q antibodies. (B) Phosphorylation of endogenous Daxx upon DNA damage in H1299 cells treated with ATM siRNA or control siRNA. (C) ATM phosphorylates Daxx at Ser564 *in vitro.* Top two panels: phosphorylated Daxx was detected by autoradiography (^32^P-Daxx) and western blot (pS564-Daxx). Bottom two panels: input of ATM and Daxx proteins were analyzed by western blot and Coomassie Blue staining, respectively. (D) H1299 cells transfected with the GFP-Daxx were untreated (0) or treated with ETP for 1 or 4 h. Endogenous PML was detected by anti-PML antibody and Texas Red-labeled secondary antibody. Images were captured using confocal microscopy. (E) H1299 cells were transfected with Flag-Daxx or Flag-Daxx S564A. Cells were stained with anti-Flag and anti-PML antibodies.

To investigate whether ATM is the kinase responsible for Daxx phosphorylation at Ser564, we knocked down ATM using siRNA. This diminished the phosphorylation of both exogenous and endogenous Daxx upon DNA damage (Figure 3, A and B). To determine whether ATM directly phosphorylates Daxx at Ser564, we performed an *in vitro* kinase assay with various purified recombinant ATM and Daxx proteins in the presence of γ-^32^P-ATP. Daxx was then analyzed by autoradiography, as well as by western blot using anti-pSer564-Daxx antibody. Daxx was phosphorylated *in vitro* by wild-type ATM, but not by a kinase-dead (KD) ATM mutant (Figure 3C, lanes 1 vs. 2). The small amount of pS564-Daxx signal in the reaction containing ATM KD (lane 2) was likely due to phosphorylation that pre-existed on the Daxx protein purified from HEK293 cells, because minimal ^32^P incorporation of Daxx was detected in this reaction. Compared to Daxx, ATM-mediated phosphorylation of Daxx S564A was weakly detected by ^32^P incorporation (lane 3), but not by anti-pS564 antibody (lane 4). This weak phosphorylation likely occurred on an amino acid residue(s) distinct from S564. Together, these data indicate that ATM can phosphorylate Daxx directly and mainly at Ser564.

Under certain stress conditions such as glucose deprivation, Daxx translocates from the nucleus to the cytoplasm [25]. However, upon DNA damage the partition of Daxx in cellular compartments remained unchanged (Figure 3D). Additionally, Daxx S564A showed a cellular localization pattern similar to that of wild-type Daxx, which accumulated in promyelocytic leukemia protein (PML) nuclear bodies (Figure 3E). These results suggest that DNA damage-induced phosphorylation of Daxx does not alter its localization.

### Phosphorylation of Daxx at Ser564 regulates its interaction with Mdm2

**Figure 4:**
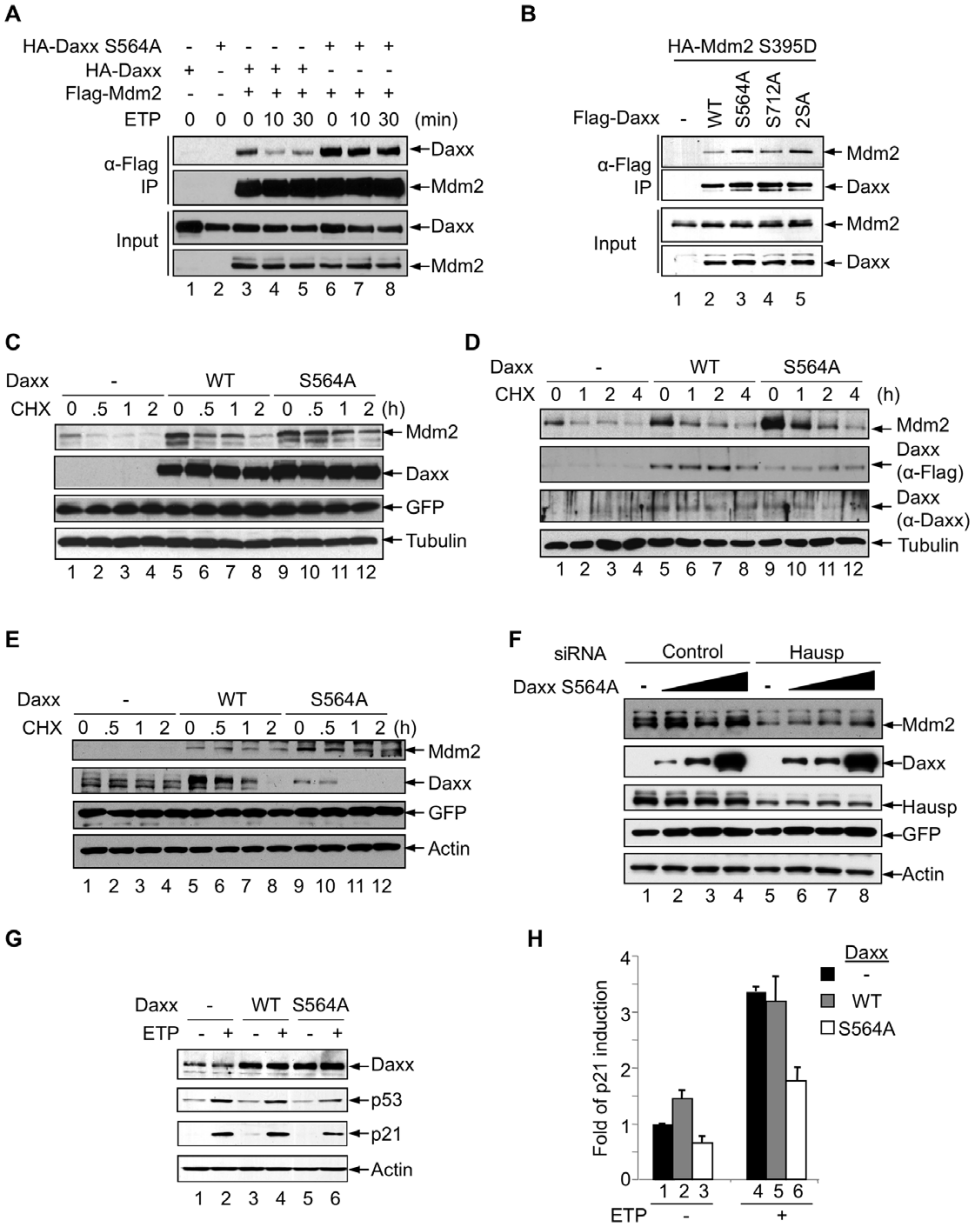
Phosphorylation of Daxx at Ser564 regulates its interaction with Mdm2. (A) Daxx S564A has enhanced binding to Mdm2 upon DNA damage. H1299 cells were transfected with either HA-Daxx or HA-Daxx S564A alone, or together with Flag-Mdm2. Cells were treated with MG-132 (20 μM) for 4 h and ETP (20 μM) for the indicated times. Cell lysates were incubated with M2 beads. Input and immunoprecipitated proteins were analyzed by western blot. (B) HA-Mdm2 S395D was transfected alone and together with the indicated Flag-tagged Daxx plasmids into *p53-/- Mdm2-/-* MEFs. Cell lysates were incubated with M2 beads. The input lysates and immunoprecipitated proteins were analyzed by western blotting. (C) H1299 cells were transfected with HA-Mdm2 alone or together with Flag-Daxx or Flag-Daxx S564A. Cells were treated with CHX (50 μg/ml) for the indicated time periods and subjected to western blot analysis. Tubulin and co-transfected GFP are shown as controls for sample loading and transfection efficiency, respectively. (D) Daxx S564A can prolong the half-life of endogenous Mdm2. U2OS cells stably expressing Flag-Daxx or Flag-Daxx S564A were treated with CHX (20 μg/mL) for the indicated time. The expression of Daxx was detected separately by anti-Daxx and anti-Flag antibodies. (E) Daxx S564A can prolong the half-life of Mdm2 upon DNA damage. Flag-Mdm2 was transfected alone or together with the indicated HA-tagged Daxx plasmids into *p53-/- Mdm2-/-* MEFs. Cells were treated with ETP (20 μM) for 1 h prior to cycloheximide (CHX, 50 μg/ml) treatment for indicated time. GFP and actin are shown as controls for transfection and sample loading, respectively. (F) The effect of Daxx S564A on Mdm2 is dependent upon Hausp. Increasing amounts of Flag-Daxx S564A were transfected into U2OS cells treated with either control or Hausp siRNA and analyzed by western blot. Actin and GFP are shown as controls for sample loading and transfection efficiency, respectively. (G, H) Daxx S564A inhibits p53-mediated gene expression. MCF-7 (G) and U2OS (H) cells stably expressing Flag-Daxx or Flag-Daxx S564A were treated with 10 μM ETP for 8 h. Protein (G) and RNA (H) expression was analyzed by western blot and quantitative RT-PCR, respectively.

Next we investigated whether phosphorylation by ATM influences the interaction between Daxx and Mdm2. A co-immunoprecipitation assay showed that in comparison to wild-type Daxx, Daxx S564A interacted with Mdm2 much more strongly (Figure 4A, lanes 3 vs. 6). As expected [20], wild-type Daxx rapidly dissociated from Mdm2 upon DNA damage (lanes 4 and 5). In contrast, the majority of the Daxx S564A protein remained bound to Mdm2 under the same condition (lanes 7 and 8), suggesting that the lack of Ser564 phosphorylation on Daxx enhances its interaction with Mdm2 and increases Mdm2 stability. Because ATM phosphorylates Mdm2 on S395 [22], we evaluated the affect of Daxx and Daxx S564A on a phospho-mimic Mdm2 mutant (S395D). Compared to Daxx, Daxx S564A showed a stronger interaction with Mdm2 S395D (Figure 4B, lanes 3 vs. 2), suggesting that the lack of Ser564 phosphorylation also augments the interaction of Daxx with the phosphorylated form of Mdm2.

A previous study reported that Daxx is phosphorylated at S712, in response to DNA damage, by the ATM/ATR signaling network [26]. We tested whether this phosphorylation event impacts the Daxx-Mdm2 interaction. Daxx S712A and wild-type Daxx showed a similar level of interaction with Mdm2 S395D (Figure 4B, lanes 4 vs. 2). Additionally, Daxx S564A/S712A (2SA) and Daxx S564A showed comparable interactions with Mdm2 S395D (lanes 5 vs. 2). These results suggest that the phosphorylation of Daxx at Ser564, but not at Ser712, is the main event that regulates its interaction with Mdm2.

To examine the effect of Daxx phosphorylation on Mdm2 stability, we expressed Mdm2 alone, together with Daxx, or together with Daxx S564A in cells, and examined the half-life of the Mdm2 protein using cycloheximide to inhibit new protein synthesis. As expected, Daxx increased the expression levels of Mdm2 and prolonged Mdm2 half-life (Figure 4C, lanes 5-8 vs. 1-4). However, compared to Daxx, Daxx S564A elevated Mdm2 to higher levels and further stabilized it (lanes 9-12 vs. 5-8). To examine the effect of Daxx S564A on endogenous Mdm2, we used retroviral infection to establish U2OS cell lines stably expressing Daxx or Daxx S564A. Compared to Daxx, Daxx S564A shows a stronger ability to increase steady-state levels and the half-life of endogenous Mdm2 (Figure 4D). To determine if Daxx S564A increases Mdm2 stability upon DNA damage, we expressed Mdm2 together with either Daxx or Daxx S564A, and treated the cells with etoposide and then with cycloheximide. Mdm2 stability was enhanced in the presence of wild-type Daxx, compared to its absence (Figure 4E, lanes 5-8 vs. 1-4). Of note, Mdm2 stability was enhanced even more in the presence of Daxx S564A as compared to in the presence of Daxx, despite Daxx S564A being expressed at levels lower than Daxx (Figure 4E, lanes 9-12 vs. 5-8). Thus, preventing ATM-mediated Daxx phosphorylation leads to the stabilization of Mdm2 in cells harboring DNA damage.

The stabilization of Mdm2 by Daxx is dependent upon Hausp [20]. To confirm that Hausp is also necessary for the Daxx S564A-mediated effect on Mdm2 levels, we treated U2OS cells with a Hausp siRNA or a control siRNA and expressed increasing amounts of Daxx S564A (Figure 4F). Endogenous Mdm2 stability is enhanced in the presence of Daxx S564A, but the effect is greatly diminished in Hausp siRNA-treated cells (Figure 4F, lanes 5-8 vs. 1-4).

We next examined whether ATM-mediated Daxx phosphorylation regulates p53 function. DNA damage-induced p53 activation was noticeably impaired in Daxx S564A-expressing cells compared to Daxx-expressing cells (Figure 4G). Consequently, the induction of the p53 target gene p21 was also impaired in Daxx S564A-expressing cells (Figure 4, G and H). Together, these results suggest that ATM-mediated Daxx phosphorylation contributes to Mdm2 destabilization and p53 activation upon DNA damage.

## Discussion

Prompt and precise activation of p53 in response to DNA damage is crucial for maintaining genomic stability. The accumulation of p53 requires the inactivation of the principal p53 antagonist Mdm2; however, the underlying mechanism for Mdm2 inactivation is not well understood. The function of Mdm2 is critically dependent on Daxx, which prevents Mdm2 degradation and also enhances E3 activity of Mdm2 towards p53. Daxx is separated from Mdm2 by DNA damage signals. This current study suggests that DNA damage signals result in Daxx phosphorylation at Ser564 by ATM. This phosphorylation event likely causes the disassembly of the Mdm2-Daxx complex, leading to Mdm2 inactivation and sequential enhancement of p53 activity.

ATM mediates the DNA damage response via phosphorylation of a large number of substrates. In the p53 pathway, both p53 itself and Mdm2 are ATM targets. The identification of Daxx as another ATM target supports the notion that ATM modulates the same pathway at various entry points to elicit robust, yet fine-tuned, responses. A previous study using proteomic analysis identified Daxx Ser712 as an ATM target site in response to DNA damage [26]. However, this phosphorylation does not appear to affect the interaction between Daxx and Mdm2 (Figure 4). During the early stages of DNA damage response, Daxx is also separated from Hausp [20]. However, mutations that block Daxx phosphorylation at Ser564 do not affect the Daxx-Hausp interaction (data not shown). It may be that a still unidentified phosphorylation event(s) on Hausp is also required for the dissociation of Daxx and Hausp. The dynamics of the Mdm2-Daxx-Hausp complex underscore its importance in the p53 pathway. As the central component of this complex that links Mdm2 with Hausp, Daxx appears to be a focal point for the regulation of p53. It may be a promising target for selectively reactivating p53 in p53-wild-type tumor cells through a non-genomic way.
