# Correction: Phosphorylation of Daxx by ATM Contributes to DNA Damage-Induced p53 Activation

**DOI:** 10.1371/annotation/032b026d-5bcf-4c1b-aa15-65c226a53818

**Published:** 2013-09-19

**Authors:** Jun Tang, Trisha Agrawal, Qian Cheng, Like Qu, Michael D. Brewer, Jiandong Chen, Xiaolu Yang

The following grant was missing from the Funding section: T.A. was a predoctoral appointee of a National Cancer Institute training grant (T32CA009140).

